# Overview of Artificial Intelligence–Driven Wearable Devices for Diabetes: Scoping Review

**DOI:** 10.2196/36010

**Published:** 2022-08-09

**Authors:** Arfan Ahmed, Sarah Aziz, Alaa Abd-alrazaq, Faisal Farooq, Javaid Sheikh

**Affiliations:** 1 AI Center for Precision Health Weill Cornell Medicine-Qatar Doha Qatar; 2 Center for Digital Health and Precision Medicine Qatar Computing Research Institute Doha Qatar

**Keywords:** diabetes, artificial intelligence, wearable devices, machine learning, mobile phone

## Abstract

**Background:**

Prevalence of diabetes has steadily increased over the last few decades with 1.5 million deaths reported in 2012 alone. Traditionally, analyzing patients with diabetes has remained a largely invasive approach. Wearable devices (WDs) make use of sensors historically reserved for hospital settings. WDs coupled with artificial intelligence (AI) algorithms show promise to help understand and conclude meaningful information from the gathered data and provide advanced and clinically meaningful analytics.

**Objective:**

This review aimed to provide an overview of AI-driven WD features for diabetes and their use in monitoring diabetes-related parameters.

**Methods:**

We searched 7 of the most popular bibliographic databases using 3 groups of search terms related to diabetes, WDs, and AI. A 2-stage process was followed for study selection: reading abstracts and titles followed by full-text screening. Two reviewers independently performed study selection and data extraction, and disagreements were resolved by consensus. A narrative approach was used to synthesize the data.

**Results:**

From an initial 3872 studies, we report the features from 37 studies post filtering according to our predefined inclusion criteria. Most of the studies targeted type 1 diabetes, type 2 diabetes, or both (21/37, 57%). Many studies (15/37, 41%) reported blood glucose as their main measurement. More than half of the studies (21/37, 57%) had the aim of estimation and prediction of glucose or glucose level monitoring. Over half of the reviewed studies looked at wrist-worn devices. Only 41% of the study devices were commercially available. We observed the use of multiple sensors with photoplethysmography sensors being most prevalent in 32% (12/37) of studies. Studies reported and compared >1 machine learning (ML) model with high levels of accuracy. Support vector machine was the most reported (13/37, 35%), followed by random forest (12/37, 32%).

**Conclusions:**

This review is the most extensive work, to date, summarizing WDs that use ML for people with diabetes, and provides research direction to those wanting to further contribute to this emerging field. Given the advancements in WD technologies replacing the need for invasive hospital setting devices, we see great advancement potential in this domain. Further work is needed to validate the ML approaches on clinical data from WDs and provide meaningful analytics that could serve as data gathering, monitoring, prediction, classification, and recommendation devices in the context of diabetes.

## Introduction

### Background

Diabetes, also known as diabetes mellitus, is a metabolic disease characterized by elevated blood glucose levels, which can ultimately result in many complications such as heart attack, stroke, kidney failure, leg amputation, vision loss, and nerve damage [1]. As the world embarks on a centennial anniversary since the development of insulin to manage glucose levels of people with diabetes, we have seen remarkable advances during these 100 years, with improved life expectancy and quality of life [2]. Noncommunicable diseases such as metabolic syndrome and diabetes continue to be among the leading causes of disability and mortality [3]. The number of cases and their prevalence have steadily increased over the last few decades. According to the World Health Organization, 1.5 million people died in 2012 alone because of diabetes, with an additional 2.1 million deaths caused by a higher than optimal blood glucose level, resulting in increased risks of cardiovascular and other diseases. A total of 463 million people, globally, were affected by type 2 diabetes (T2D) mellitus in 2019. Furthermore, it is predicted that 700 million individuals would develop diabetes by 2045 [4]. Although the World Health Organization acknowledges that there is no one fixed solution and that a coordinated multicomponent intervention is needed, it outlines technology as one of the key stakeholders in reducing the impact of diabetes in addition to input from governments, health care providers, people with diabetes, civil society, food producers and manufacturers, and suppliers of medicine [1].

Despite the advancements in blood glucose monitoring techniques, the mainstream detection technology remains largely invasive. The commonly used home electronic glucometers involve people with diabetes invasively self-pricking to draw blood from fingertips, opening them up to infections as well as stress and pain caused by the procedure that is often expected multiple times a day.

The availability and advancements of smart devices, such as smartphones, have made the monitoring of diabetes-related features more accessible. Many studies have examined this much welcomed technology [5,6]. These normally require the use of an external attachable sensor, and monitoring is then delivered via an app or a separate continuous glucose monitoring (CGM) device, which can still be semi-invasive and require a connection range via Bluetooth or Wi-Fi signals. The use of completely noninvasive technology in the form of wearable devices (WDs) for regulating and monitoring glucose levels for people with diabetes is a fairly new concept and is in its infancy. Commercially available devices, such as smart watches and smart bands, can take measurements using sensors that researchers have reported on their usefulness in diabetes monitoring [7,8]. Such technologies can be affordable and easily accessible, and when used properly, can improve the quality of life of patients in a noninvasive manner. With their widespread commercial use and acceptance owing to their fashionable nature, globally researchers have a unique opportunity to provide medical care away from hospital settings and bulky invasive hardware in an affordable manner without requiring expert assistance. WDs have an increasing capacity, although not at the level of smartphones, to gather, store, transmit, and process data; the features can then be used for management, treatment, assessment, and sometimes even prediction. Furthermore, many WDs are normally connected via Wi-Fi or Bluetooth to external devices, such as a smartphone, where computationally expensive processing is performed for the simple purpose of storage or as a gateway to cloud spaces. Cloud storage can facilitate monitoring by clinicians without the need of hospitalization. Several useful sensors already exist incorporated into WDs similar to those of smartphones, including electrocardiogram (ECG), photoplethysmography, galvanic skin response, near infrared, and accelerometer sensors. WDs have additional advantages when it comes to sensing physiological signs, such as heart rate, ECG, and skin temperature. This is largely owing to their close contact with the wearer, which is of particular interest when monitoring diabetes-related metrics.

Artificial intelligence (AI) is a broader term that encompasses machine learning (ML). Technically, ML is a subset of AI, often loosely used interchangeable buzzwords. As a high-level definition, AI is anything related to making machines smarter (eg, computational search algorithms). ML, on the other hand, is an AI system that can self-learn via an algorithm, and as a result, such a system becomes smarter without human intervention over time (eg, classifying an outcome) [9]. Deep learning, on the other hand, is another branch of AI that attempts to mimic the human brain in terms of how it processes large amounts of data and has already shown success rates in areas such as diabetic retinopathy screening [10]. ML principles have been applied in clinical settings to build algorithms to support predictive models for the risk of development of diabetes [11]. AI has also been shown to provide useful management tools to deal with large amounts of data [12]. Owing to the large amount of data measurable through continuous monitoring via wearables, AI can be used to further analyze the acquired data. This can help to understand and draw meaningful information from the gathered data and provide advanced and clinically meaningful analytics. Many researchers have adapted existing WDs not originally intended for diabetes management and adapted the sensory information for use in diabetes-related metrics, and some have created prototypes especially designed for diabetes [13,14]. WDs are used for a variety of reasons, including monitoring, prevention, glucose estimation, diagnostics, classification, and prevention, but the number of studies that are reported are low in comparison with those that make use of smartphones for example. With the increased potential outreach of WDs globally, especially when combined with the ever-expanding field of AI-incorporating ML algorithms, the correct management of large amounts of data and processing with ML algorithms holds great potential for quality-of-life improvement in people with diabetes [15].

### Research Problem and Aim

Many studies have been conducted on AI-based WDs for diabetes. Exploring the features of AI-based WDs reported in these studies is important for developers, patients, health care providers, and researchers to identify the recent advances and challenges in this area. Although several reviews were conducted in this area, (1) they were focused on smartphones and AI for diabetes [16-18], (2) they were focused on WDs in general rather than AI-based WDs [17,19], and (3) they did not summarize the features of AI-based WDs in a thorough manner [16-19]. Therefore, we aimed to explore the features of AI-based WDs for diabetes as reported in previous studies. We believe that this review will allow developers and researchers to advance further in this field by highlighting the gaps and opportunities.

## Methods

### Overview

This scoping review was carried out to satisfy this study’s goals of exploring features of AI-driven wearable technologies for diabetes. In order to construct a complete scoping review, the *PRISMA*-*ScR* (Preferred Reporting Items for Systematic Reviews and Meta-Analyses extension for Scoping Reviews) [20] was used as a guiding approach. The PRISMA-ScR checklist is shown in Multimedia Appendix 1.

### Search Strategy

#### Search Sources

The article search for this review began by identifying all relevant studies using 7 electronic databases: MEDLINE, PsycINFO, EMBASE, IEEE Xplore, ACM Digital Library, Web of Science, and Google Scholar. We scanned the first 100 hits retrieved by searching Google Scholar. The reason being Google Scholar typically returns several items that are sorted by relevance to the search topic. Bibliographic collection was conducted from October 25 to October 30, 2021. The reference lists of the included articles were then searched for additional sources. We also checked relevant articles that cited the included studies using Google Scholar’s “cited by” tool (forward reference list checking).

#### Search Terms

A number of different sets of keywords were designed to search databases depending on each database’s search term limit; as IEEE and Google Scholar have term limits, search queries were truncated based on the required limit. We considered the research topics included in the database to complete our search queries. We combined *Diabetic* OR *Diabetes* keywords describing the relevant population (people with diabetes), with each kind of relevant intervention to wearables (wearable* OR smart watch* OR smart* OR smartwatch* OR fitness band* OR flexible band* OR wristband* OR smart insole* OR bracelet*) and AI (Artificial Intelligence OR Machine Learning OR Deep Learning OR Decision tree OR K-Nearest Neighbor* OR Support vector machine* OR Recurrent neural network* OR convolutional neural network* OR Artificial neural network* OR Naïve Bayes OR Naive Bayes OR Fuzzy Logic OR K-Means OR Random Forest OR LSTM OR autoencoder OR boltzmann machine OR deep belief network). For example, the following search terms were applied in Google Scholar: (Artificial Intelligence OR Machine Learning OR Deep Learning OR convolutional neural network* OR Artificial neural network*) AND (wearable* OR smart watch* OR smart*) AND (Diabetic OR Diabetes). All the databases had search time period criteria that were enabled and set with the search query from 2015 to present; in addition, the language checkbox in each database was set to English only. Full search terms for each electronic database searched are available in Multimedia Appendix 2.

Studies were chosen based on the criteria in Textbox 1. Peer-reviewed articles and published protocols were included only if they were related to wearables that could be used by an individual outside of a clinical setting. They also had to use AI for the purpose of diabetes and be classified as noninvasive. For full inclusion and exclusion criteria refer to Textbox 1.

**Inclusion and exclusion criteria.**

**Inclusion criteria**

1. Publications that are in the English language.
2. Peer-reviewed articles including proposals.
3. Population with or suspected to have diabetes. No restrictions regarding their age, gender, and ethnicity.
4. Commercial, medical, or prototypes but with condition wearable device and uses artificial intelligence (AI).
5. Wearable usable by individual person not with help of clinical staff or plugged in to hospital setting.
6. Wearables using methods for diabetes analysis are to be noninvasive.

**Exclusion criteria**

1. Any study that does not contain AI as an intervention.
2. People with other diseases, health care providers, and caregivers as population.
3. Not a wearable device (example artificial implant or body infused).
4. Studies opting statistical measures only, for analysis of collected data.
5. Sensors or tracking devices infused inside a person’s body.
6. Wearable devices that need professional sittings or hospital sittings.

#### Study Selection

This review’s studies were selected in 2 steps. In the first stage, 2 reviewers (AA and SA) independently reviewed the titles and abstracts of all retrieved papers. In the second phase, the same reviewers individually read the whole texts of the papers included in the first step. Rayyan (Qatar Computing Research Institute, Hamad Bin Khalifa University) [21], a web-based tool developed for data management for systematic and scoping reviews, was used to upload all the articles acquired from databases in a Research Information Systems format; then, filtering and citations were managed. During the first and second steps of the selection process, any disagreements between the 2 reviewers were resolved through conversation and decisions were made based on consensus.

### Data Extraction

AA and SA constructed the data extraction form, as shown in Multimedia Appendix 3. The data extraction technique was carried out independently by 2 reviewers (AA and SA), and any discrepancies were resolved by discussion and consensus. Microsoft Excel was used to record the data extracted.

### Data Synthesis

SA synthesized the extracted data using the narrative approach, aggregating the data using tables and text and nonstatistical techniques. For being more precise, we presented the search results followed by general features of the studies, finally describing characteristics of the WDs and AI technologies. We described the general features of WDs (eg, device placement, type, and operating system [OS]) and their technical features (ie, features of sensors, such as sensors used, sensing approach, and primary measurements). The AI features were addressed based on the models used, the evaluation metrics, and their applications.

## Results

### Search Results

**Figure 1:**
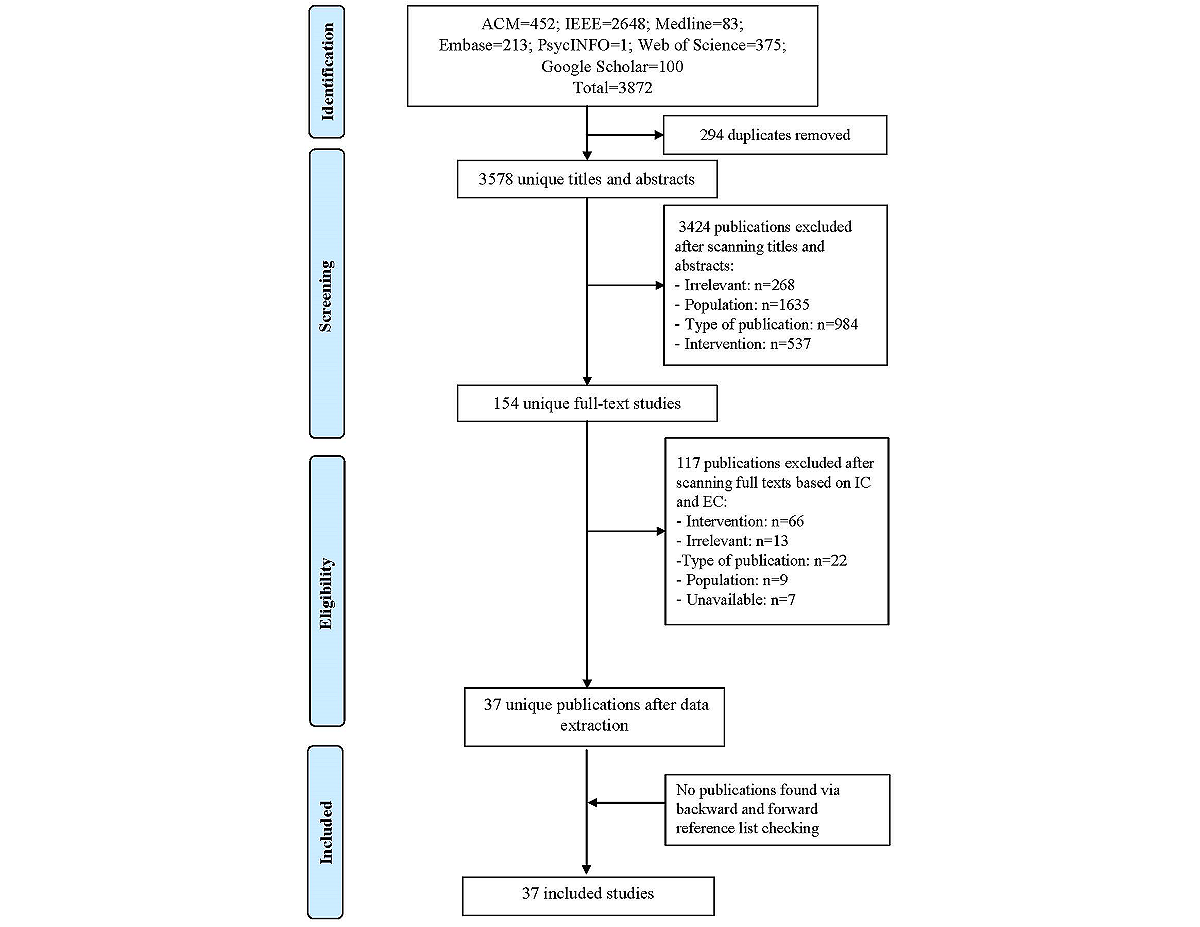
PRISMA (Preferred Reporting Items for Systematic Reviews and Meta-Analyses) flow chart of the study selection process. EC: exclusion criteria; IC: inclusion criteria.

Having searched 7 bibliographic databases, this study returned 3872 citations. As shown in Figure 1, a total of 294 duplicates were subsequently removed, leaving 3578 unique titles, and abstracts; publications that did not make use of AI technologies via WDs for diabetes management were considered irrelevant. Of these, we further excluded 3424 citations after screening their titles and abstracts. Of the remaining 154 references, 117 publications were excluded during the full-text screening. We were left with 37 studies, and this number remained unchanged even after performing backward and forward reference list checking. The synthesis included a total of 37 articles (Multimedia Appendix 4 [7,8,13,14,22-54]).

### General Description of Included Studies

Table 1 highlights the general features of the studies included. Many of the included studies (27/37, 73%) were published between 2019 and 2021, with the remaining 27% (10/37) published between 2016 and 2018. Most of the included studies were published in IEEE (21/37, 57%). A large proportion of the studies were authored by institutes in the United States (7/37, 19%), China (5/37, 14%), and India (5/37, 14%). A total of 26 of the 37 studies (70%) were journal articles, and the remainder were conference proceedings (11/37, 30%). Most of the studies targeted type 1 diabetes (T1D), T2D, or both (21/37, 57%), whereas 32% (12/37) did not specify the type of diabetes and mentioned diabetes in general. The remainder targeted prediabetes or a combination of T1D, T2D, and prediabetes (4/37, 11%). Features of each included study are shown in Multimedia Appendix 5.

**Table 1:** General features of included studies (n=37).

Features		Studies, n (%)	Study ID
**Year**			
	2019	10 (27)	S4, S8, S10, S16, S18, S20, S21, S24, S29, S30
	2020	9 (24)	S3, S7, S11, S13, S15, S17, S19, S22, S35
	2021	8 (22)	S5, S9, S12, S14, S25, S27, S28, S33
	2018	6 (16)	S1, S6, S23, S34, S36, S37
	2017	3 (8)	S2, S26, S32
	2016	1 (3)	S31
**Publisher**			
	IEEE	21 (57)	S1, S3, S5, S9-S11, S13-S18, S20, S24, S26, S28, S29, S31, S32, S36, S37
	Elsevier	3 (8)	S2, S12, S22
	MDPI	3 (8)	S6-S8
	ACM	2 (5)	S21, S35
	Other (JMIR, IET, ICST, Confluence, BMJ Publishing Group, SPIE, Telemedicine and e-Health, SAGE)	8^a^ (22)	S4, S19, S23, S25, S27, S30, S33, S34
**Country**			
	United States	7 (19)	S13, S14, S21, S27, S30, S31, S34
	China	5 (14)	S5, S15, S18, S19, S37
	India	5 (14)	S12, S17, S23, S25, S32
	Pakistan	2 (5)	S3, S20
	Switzerland	2 (5)	S6, S35
	Bangladesh	2 (5)	S10, S24
	Other (Korea, Colombia, Canada, Morocco, Mexico, Italy, Macedonia, Sri Lanka, United Kingdom, Russia, Taiwan, Philippines, Saudi Arabia, Germany)	14^b^ (38)	S1, S2, S4, S7, S8, S9, S11, S16, S22, S26, S28, S29, S33, S36
**Publication type**			
	Journal articles	26 (70)	S1-S20, S22, S23, S27, S28, S33, S34
	Conference proceedings	11 (30)	S21, S24-S26, S20-S32, S35-S37
**Diabetes type studied**			
	Both T1D^c^ and T2D^d^	9 (24)	S1, S5, S6, S8, S10, S11, S14, S24, S36
	T2D	7 (19)	S2-S4, S15, S16, S21, S29
	T1D	5 (14)	S13, S22, S30, S34, S35
	T1D, T2D, and prediabetes	2 (5)	S12, S25
	T1D and prediabetes	1 (3)	S17
	Prediabetes	1 (3)	S27
	Not specified	12 (32)	S7, S9, S18, S19, S20, S23, S26, S28, S31, S32, S33, S37

^a^1 study for each publication.

^b^1 study for each country.

^c^T1D: type 1 diabetes.

^d^T2D: type 2 diabetes.

### Study Design Features

Table 2 outlines details about the studies associated with this review. More than half of the studies (21/37, 57%) had the aim of estimation and prediction of glucose (10/37, 27%) or glucose level monitoring (11/37, 30%). A couple of studies had multiple aims, and the remainder aimed to provide diabetes classification (4/37, 11%), diagnostic solutions (5/37, 14%), self-administration and monitoring (4/37, 11%), and prevention (2/37, 5%). Most of the studies did not mention anything about security (31/37, 84%); the remainder did specify security measures taken (6/37, 16%). Participants’ demographics depicted most of them being adult (18/37, 49%) with both genders considered in equal proportional in most of the studies (10/37, 27%). Approximately 41% (15/37) of the studies used diverse populations by separating them into people with diabetes and people without diabetes. Study design features of each included study are shown in Multimedia Appendix 5.

**Table 2:** Study design features (n=37).

Features				Studies, n (%)		Study ID
**Study aim**						
	Blood glucose estimation (predictions)		10 (27)		S3, S18, S19, S21, S23, S25, S27, S28, S32, S33, S37	
	Glucose level monitoring		10 (27)		S7-S11, S15, S20, S24, S26, S30	
	Diagnostic solution		5 (14)		S4, S29, S33, S34, S35	
	Diabetes classification		4 (11)		S12-S14, S17	
	Self-administration and monitoring		4 (11)		S1, S5, S6, S31	
	Prevention		2 (5)		S2, S16	
	Other disease predictions, detection, and monitoring (hypoglycemia and foot temperature)		2 (5)		S22, S36	
**Privacy and security**						
	Not mentioned		31 (84)		S1-S22, S24-S26, S29, S30, S34-S37	
	Mentioned		6 (16)		S23, S27, S28, S31, S32, S33	
**Data source**						
	Private		25 (68)		S2, S3, S5, S7, S8-S19, S21, S22, S24, S26, S29, S34-S37	
	Public		4 (11)		S1, S4, S6, S25	
	Not mentioned		2 (5)		S20, S30	
**Participant demographics**						
	**Age group (years)^a^**					
		Children and young adults (≤18)	1 (3)		S8	
		Adult (19-65)	18 (49)		S2-S5, S8, S10, S13, S15, S16, S17, S19, S21, S22, S27, S29, S31, S33, S34	
		Older adult (>65)	6 (16)		S2, S4, S15, S21, S22, S33	
		Not mentioned	19 (51)		S1, S6, S7, S9, S11, S12, S14, S18, S20, S23-S26, S28, S30, S32, S35-S37	
	**Gender**					
		Male	10 (27)		S2, S3, S5, S13, S15, S17, S18, S27, S29, S34	
		Female	10 (27)		S2, S3, S5, S13, S15, S17, S18, S27, S29, S34	
		Not mentioned	27 (73)		S1, S4, S6-S12, S14, S16, S19-S26, S28, S30-S33, S35-S37	
	**Diabetes^b^**					
		Yes	14 (38)		S1, S4, S5-S7, S10, S12, S14, S15, S18, S19, S21, S27, S34, S36	
		No	15 (41)		S1, S5, S6, S8-S10, S12, S14, S18, S19, S27, S29, S31, S33, S36	
		Not mentioned	17 (46)		S2, S3, S11, S13, S16, S17, S20, S22-S26, S29, S30, S32, S35, S37	

^a^Numbers do not add up as participants in some studies belong to more than one age group.

^b^Numbers do not add up as participants in some studies were diabetic and nondiabetic.

### Features of WDs

#### General Features of Wearables

Table 3 highlights the general features of WDs; some studies used multiple devices. Only 41% (15/37) of the studies used commercially available WDs, whereas 59% (22/37) used prototypes. Most of the studies (22/37, 59%) included wrist-worn devices. In most of the studies, the device type was in the form of a wearable sensor (14/37, 38%), followed by smartwatch (8/37, 22%) and smart wristband (9/37, 24%). Only one study reported a smart sock and another reported smart clothes. Among the developed wearable technologies used, Empatica E4 was the most cited (6/37, 16%), followed by Glutrac (3/37, 8%). For OSs, most of the studies reported devices either directly or indirectly compatible with iPhone OS and Android OS 43% (16/37); OS was not mentioned in a large number of studies (11/37, 28%), and 8% (3/37) mentioned Android only and 5% (2/37) mentioned iPhone OS only. For gateway (ie, a hardware that acts as a “gate” between 2 networks or any device that enables traffic to flow in and out of the network), many of the studies did not mention any sort of gateway (17/37, 46%). Most of the studies that mentioned a gateway used a smartphone (16/37, 43%). Host devices (devices where the actual manipulation of collected data was performed, ie, processing) were used in many of the studies, the most popular being cloud-based (18/37, 49%), many did not report any use of a host device (8/37, 22%), and smart devices were mentioned in 16% (6/37) of studies. For the purpose of transferring data (mode of data transfer) from the WD, the majority of devices reported Bluetooth as the means of transfer (19/37, 51%); 19% (7/37) of studies did not mention the mode of transfer. A total of 16% (6/37) of studies reported the use of some form of internet connection as the mode of transfer (ie, Wi-Fi or mobile network). Features of WDs for each included study are shown in Multimedia Appendix 5.

**Table 3:** General features of wearable devices (n=37).

Features		Studies, n (%)	Study ID
**Technology status**			
	Prototype	22 (59)	S1, S3-S5, S8-S11, S16, S17, S20, S23, S24, S26, S28-S33, S36, S37
	Commercial	15 (41)	S2, S6, S7, S12-S15, S18, S19, S21, S22, S25, S27, S34, S35
**Device type**			
	Smart clothes	1 (3)	S1
	Smart socks	1 (3)	S31
	Smart watch	8 (22)	S2, S7, S14, S15, S18, S19, S28, S35
	Smart watch and wearable sensor	2 (5)	S21, S24
	Smart wristband	9 (24)	S4, S6, S12, S13, S25, S27, S30, S33, S34
	Smart wristband, smart footwear, and smart neckband	2 (5)	S23, S32
	Wearable sensor	14 (38)	S3, S5, S8-S11, S16, S17, S20, S22, S26, S36, S37
**Placement**			
	Body	1 (3)	S1
	Chest	1 (3)	S11
	Finger	5 (14)	S3, S8, S17, S20, S26
	Foot	6 (16)	S5, S9, S16, S29, S31, S36
	Hand	1 (3)	S10
	Wrist	18 (49)	S4, S6, S7, S12-S15, S18, S19, S24, S25, S27, S28, S30, S33-S35, S37
	Wrist and arm	1 (3)	S21
	Wrist or thigh	1 (3)	S2
	Wrist, foot, and neck	2 (5)	S23, S32
	Arm and body	1 (3)	S22
**Device technology^a^**			
	Actigraph	1 (3)	S21
	Arduino Nano	1 (3)	S24
	Basis Peak	1 (3)	S34
	FreeStyle Libre Flash	2 (5)	S22, S35
	Medtronic Zephyr	1 (3)	S22
	Dexcom G4 Platinum (Professional)	1 (3)	S21
	Empatica E4	6 (16)	S12, S13, S14, S25, S27, S35
	Glutrac	3 (8)	S15, S18, S19
	Mi band 2	1 (3)	S6
	Raspberry Pi Zero	2 (5)	S8, S16
	Pebble	1 (3)	S2
	Custom	2 (5)	S26, S28
	Not mentioned	18 (49)	S1, S3-S5, S7, S9-S11, S17, S20, S23, S29-S33, S36, S37
**Operating system^b^**			
	Android	3 (8)	S2, S8, S16
	iOS^c^	2 (5)	S9, S11
	Microsoft	1 (3)	S31
	Raspberry Pi OS^d^	1 (3)	S24
	iOS and Android	16 (43)	S6, S7, S12-S15, S17-S20, S22, S23, S26, S28, S30, S32
	Any desktop OS	3 (8)	S25, S27, S29
	Any smartphone OS	1 (3)	S29
	Not mentioned	11 (30)	S1, S3-S5, S10, S21, S33-S37
**Gateway**			
	Smartphone	16 (43)	S1, S6, S7, S11-S15, S17-S20, S23, S28, S30, S32
	Database servers (Hbase and Hadoop or Spark)	1 (3)	S33
	Adapter	1 (3)	S4
	Smartphone or PC	2 (5)	S25, S27
	None	17 (46)	S2, S3, S5, S8-S10, S16, S21, S22, S24, S26, S29, S31, S34-S37
**Host device**			
	Cloud (MongoDb, Database server, Google)	18 (49)	S1, S6, S7, S11-S15, S17-S19, S23, S25, S27, S28, S30, S32, S33
	PC (laptop, desktop, or Microsoft Surface)	4 (11)	S4, S20, S29, S31
	Raspberry Pi	1 (3)	S24
	Smart devices (smartphone, tablet, or PC)	6 (16)	S5, S8, S9, S16, S22, S26
	None	8 (22)	S2, S3, S10, S21, S34-S37
**Mode of data transfer**			
	Bluetooth	19 (51)	S2, S5, S6, S9, S11-S15, S18-S20, S22, S25-S28, S30, S31
	Internet (Wi-Fi or cellular or mobile network)	6 (16)	S1, S7, S8, S16, S17, S33
	Internet (Wi-Fi or cellular or mobile network) and Bluetooth	2 (5)	S23, S32
	Wired	2 (5)	S24, S29
	Removable media	1 (3)	S4
	N/A^e^	7 (19)	S3, S10, S21, S34-S37

^a^Numbers do not add up as some studies used more than one wearable device.

^b^Numbers do not add up as the WD in one study worked on 2 operating systems.

^c^iOS: iPhone operating system.

^d^OS: operating system.

^e^N/A: not applicable.

#### Technical Features of Wearables

Table 4 shows an overview of the technical features of the WDs associated with the studies in this review. Devices were often reported as having >1 device measure, and we reported these primary measures along with the measurements used for the respective studies. We observed that many studies reported blood glucose (15/37, 41%) followed by temperature (10/37, 27%), heart rate (9/37, 24%) and galvanic skin response (9/37, 24%) as their top primary device measures. Regarding the second feature shown in Table 4, the majority of the studies reported on blood glucose as the main measurement studied (27/37, 73%), followed by heart rate or variability (4/37, 11%). Most of the studies (28/37, 76%) reported an opportunistic approach (ie, no input required from the participant) when obtaining data using the WDs, whereas the remaining (9/37, 24%) used a participatory approach (ie, input required from the participants). For sensing technologies, various sensors were used, either built-in to the WD or as wearable sensors, often reported as >1 sensor per device. We observed a large number of devices in the studies reviewed reporting photoplethysmography sensor use (12/37, 32%), while optical heart rate was only seen in 5% (2/37) of studies among some of the other less-reported sensors. Features of WDs for each included study are shown in Multimedia Appendix 5.

**Table 4:** Technical features of wearables (n=37).

Feature		Studies, n (%)	Study ID
**Primary device measure^a^**			
	Blood glucose	15 (41)	S3, S8, S10, S15, S17-S22, S24, S26, S28, S30, S37
	Physiological	2 (5)	S1, S10
	Heart rate, heart rate variability, or interbeat interval of the heart	9 (24)	S6, S11, S14, S22, S23, S32-S35
	Galvanic skin response	9 (24)	S12-S14, S23, S25, S27, S32, S34, S35
	Blood volume pulse	6 (16)	S12-S14, S25, S27, S35
	Acceleration	6 (16)	S12-S14, S25, S27, S35
	Plantar pressure	5 (14)	S5, S9, S23, S29, S32, S33
	Temperature (skin, foot, shoe, air, or ambient)	10 (27)	S12, S13, S16, S23, S25, S27, S32, S34-S36
	Step count	2 (5)	S7, S16
	Other (sedentary behaviors, pulse wave information, inertial data, weight, humidity, activity patterns, frequency of food intake and water, and ankle edema quantification)	8 (22)	S2, S4, S9, S16, S21, S23, S31, S32
**Measurement studied^b^**			
	Blood glucose	27 (73)	S1, S3, S6-S8, S10-S28, S30, S33, S37
	Plantar pressure	3 (8)	S5, S9, S29
	Heart rate or heart rate variability	4 (11)	S28, S33, S34, S35
	Other (sedentary behavior, pulse wave, edema, general diabetes symptoms, temperature, sleep quality, step counts, and GSR)	7 (19)	S2, S4, S31, S32, S34-S36
**Sensing approach**			
	Opportunistic	28 (76)	S1, S2, S5, S7, S11-S14, S16-S29, S31-S33, S35-S37
	Participatory	9 (24)	S3, S4, S6, S8-S10, S15, S30, S34
**Sensing technology^c^**			
	Accelerometer	5 (14)	S2, S13, S14, S21, S27
	Photoplethysmography	12 (32)	S3, S10, S12-S15, S19, S20, S24, S25, S27, S28
	Galvanic skin response	8 (22)	S10, S13, S14, S23, S24, S27, S32, S34
	Near infrared	5 (14)	S3, S17, S18, S28, S37
	Electrocardiography	3 (8)	S11, S18, S22
	Continuous glucose monitoring	2 (5)	S21, S22
	Bluetooth	1 (3)	S6
	Pressure sensors	7 (19)	S5, S9, S23, S29, S32, S33, S36
	Infrared thermopile	3 (8)	S13, S14, S27
	Temperature sensor	6 (16)	S7, S16, S23, S24, S32, S36
	Optical heart rate sensor	2 (5)	S23, S32
	Vibration sensor and flex sensor	2 (5)	S23, S32
	Motion sensor	2 (5)	S7, S31
	Others (physiological sensors, pulse sensor, blood glucose level sensor, Raspberry Pi camera, humidity sensor, step count sensor, weight sensor, stretch sensor, and optical sensor)	6 (16)	S1, S4, S7, S8, S16, S31

^a^Numbers do not add up as WDs in many studies were used to measures many biomarkers.

^b^Numbers do not add up as some studies used more than one measure.

^c^Numbers do not add up as WDs in most studies used more than one sensor.

### Wearables Characteristics With Regard to Diabetes Measurements

#### Wearable Technology Status Versus WD Type

Multimedia Appendix 6 further visualizes the data highlighting the WD type and whether they are commercial or prototypes. Wearable sensors were the most prominent as a prototype while smartwatches and smart wristbands were the most common as commercial.

#### Diabetes Types Versus WDs

**Figure 2:**
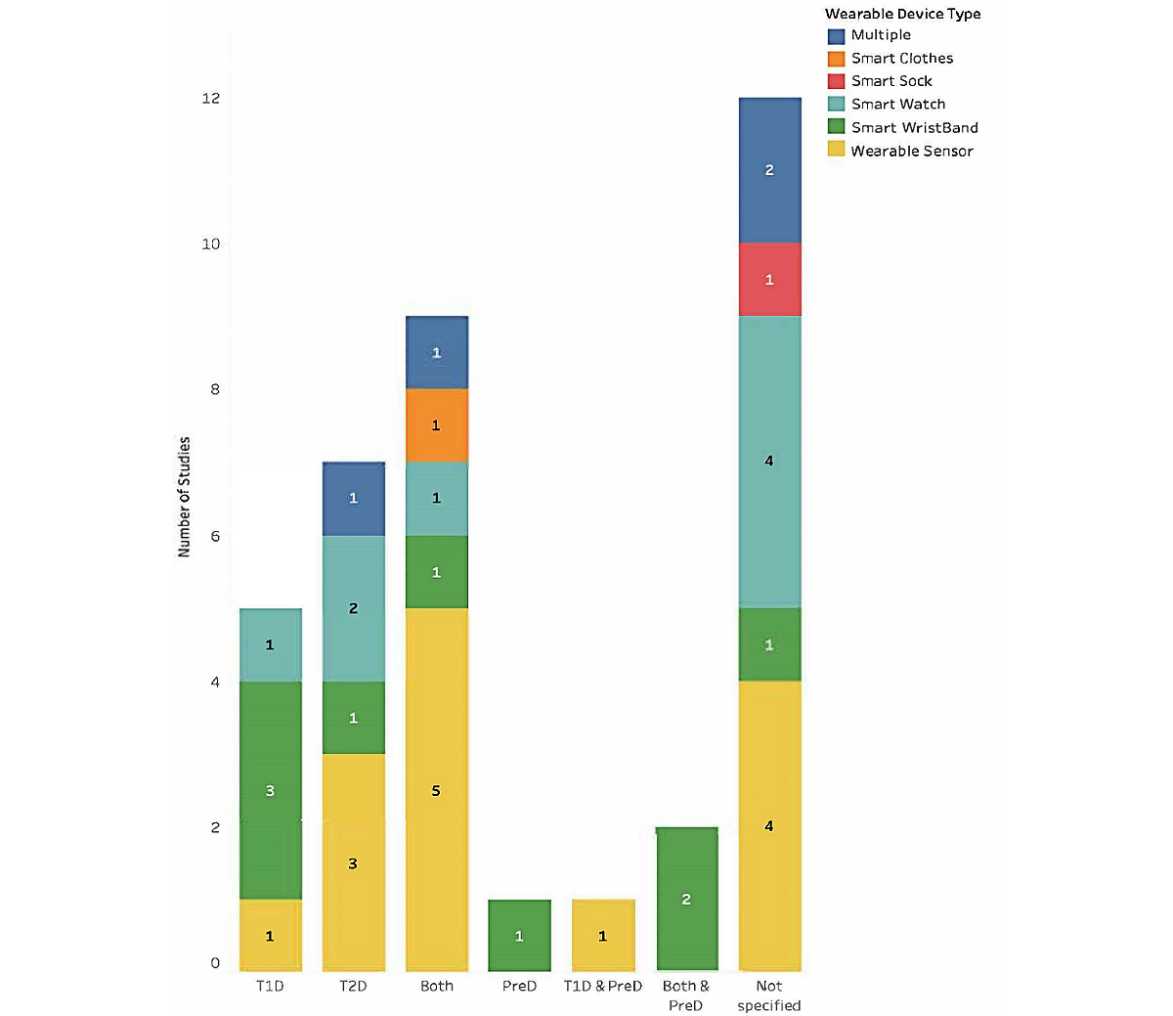
Diabetes type with regards to wearable device type. PreD: prediabetes; T1D: type 1 diabetes; T2D: type 2 diabetes.

Figure 2 shows the type of diabetes and number of studies related to each WD type. While most studies did not specify the type, T1D (as a smart wristband), T2D (as a wearable sensor), or both (as a wearable sensor or smartwatch) seem to be the most targeted types.

#### AI and ML Technologies

For the purpose of this study, we categorized the ML algorithms into 4 categories (classification models, regression models, neural network–based models, and optimization algorithms) and those that were not clearly specified by the study authors were categorized as black boxes (ie, studies that mention they make use of ML or AI but do not specify any further details of algorithms used). Many ML technologies were reported that come under these headings (refer to Table 5 for a full list), and some studies reported and compared >1 model. Support vector machine (SVM) was the most reported (13/37, 35%), followed by random forest (12/37, 32%), k-nearest neighbor (7/37, 19%), Naive Bayes (5/37, 14%), and decision trees (4/37, 11%) among the most used models from classification models. From the regression models, only linear regression (2/37, 5%) was reported in a couple of studies, whereas all others were reported by single studies only. Artificial neural networks were reported in 14% (5/37) of the studies in neural network–based models, followed by long short-term memory (4/37, 11%), convolutional neural networks (3/37, 8%), and deep neural networks (3/37, 8%); these networks were used for both classification and regression purposes. Table 5 also highlights that the majority of the studies applied the AI and ML technologies for either the purpose of blood glucose level forecasting (12/37, 32%) or classifying the participants as normal, diabetic, or prediabetic (12/37, 32%). Table 6 highlights some of the statistical measures used to evaluate the ML algorithms within the reported studies. Some studies used multiple statistical techniques for this purpose, among them were reports of accuracy (20/37, 54%) and sensitivity (9/37, 24%). While some studies did not mention which was the best ML model identified (6/37, 16%), random forest was reported as the best identified model (7/37, 19%), followed by SVM (6/37, 16%).

**Table 5:** Artificial intelligence (AI)– and machine learning (ML)–related features (n=37).

Features				Studies, n (%)		Study ID
**AI or ML technologies used^a^**						
	**Classification models**					
		Support vector machine	13 (35)		S1, S2, S4, S5, S9, S12, S13, S25, S29, S30, S33, S34, S36	
		Random forest	12 (32)		S2, S4, S5, S7, S11, S14, S15, S18, S27, S29, S36, S37	
		K-nearest neighbors	7 (19)		S5, S9, S12, S13, S25, S29, S31	
		Naive Bayes	5 (14)		S2, S7, S13, S31, S36	
		Decision tree	4 (11)		S1, S13, S31, S35	
		Ensemble learning or ensemble—boosted trees	2 (5)		S1, S13	
		Logistic regression	2 (5)		S2, S11	
		J48	2 (5)		S2, S7	
		Linear discriminant analysis or linear discriminant	2 (5)		S4, S13	
		Gradient boosting decision trees	2 (5)		S5, S35	
		AdaBoost classifier	1 (3)		S5	
		ZeroR	1 (3)		S7	
		OneR	1 (3)		S7	
		Simple logistic regression	1 (3)		S7	
		Gaussian Process classifier	1 (3)		S29	
		C4.5	1 (3)		S33	
		Linear ridge Classifier	1 (3)		S14	
		Extreme gradient boost	1 (3)		S12	
	**Regression models**					
		Linear regression	2 (5)		S3, S16	
		Support vector regression or Fine Gaussian support vector regression	1 (3)		S3	
		Random Forest regression	1 (3)		S15	
		AdaBoost regression	1 (3)		S15	
		Multilayer Polynomial regression	1 (3)		S17	
		Ensemble—boosted trees	1 (3)		S3	
		Exponential Gaussian process regression	1 (3)		S20	
	**Neural network–based models**					
		Artificial Neural Network	5 (14)		S1, S2, S8, S26, S36	
		Long short-term memory	4 (11)		S6, S13, S21, S34	
		Convolutional Neural Network	3 (8)		S10, S22, S24	
		Deep neural networks	3 (8)		S11, S13, S22	
		Recurrent Neural Network	2 (5)		S21, S34	
		Multilayer Perceptron	2 (5)		S6, S29	
	**Optimization algorithm**					
		Sequential minimal optimization	1 (3)		S7	
		L1 norm optimization	1 (3)		S19	
		Particle swarm optimization	1 (3)		S23	
	ML^a^ black box		3 (8)		S19, S23, S32	
**Application of AI technology used^b^**						
	Blood glucose level forecasting		12		S6, S8, S16, S18, S20, S22, S24, S25-28, S34	
	Blood glucose monitoring		4		11, S30, S32, S37	
	Classify patients with diabetes (normal, diabetic, and prediabetic)		12		S3, S4, S5, S6, S7, S12, S14, S21, S23, S29, S32, S36	
	Classify other diseases (patients with hypertension or hypoglycemia)		2		S33, S35	
	Evaluation of a developed system		3		S2, S10, S13	
	Feature selection		2		S3, S5	
	Performance validation		3		S1, S9, S15	
	Optimize sensors results		3		S16, S17, S19	
	Predictions for step count, shoe removal time, or serum glucose		2		S16, S17	
	Edema monitoring		1		S31	

^a^Numbers do not add up as most studies developed more than one AI algorithms.

^b^Numbers do not add up as AI algorithms in some studies were used for more than one application.

**Table 6:** Statistical evaluation of artificial intelligence and machine learning algorithm (n=37).

Characteristic		Value	Study ID
**Accuracy (%; n=20)**			
		≤80	S6, S33
		81-90	S15, S21, S28, S35, S36
		91-95	S1, S9, S13, S15, S22, S25, S29
		>95	S4, S5, S7, S12, S14, S30, S31
**Sensitivity (%; n=9)**			
		≤80	S35
		81-90	S4, S6, S25, S33
		91-95	S9, S22
		>95	S5, S7
**Specificity (%; n=7)**			
		≤85	S35
		86-90	S9, S22
		91-95	S5, S25
		>95	S4, S7
**Area under the curve (%; n=2)**			
		≤91	S22
		>91	S35
**Clarke Error Grid zone A (%; n=8)**			
		≤74	S37
		75-80	S19, S10
		81-90	S18, S28
		>90	S3, S8
		Not mentioned	S24
**Precision (%; n=6)**			
		≤80	S6, S33
		81-90	S9
		91-95	S25
		>95	S2, S7
**Root mean square error (%; n=4)**			
		<5	S19, S21
		5-15	S17
		>15	S27
**Others, n (%)**			
		8 (22)	S3, S8, S16, S17, S19, S21, S27, S37
**Best model identified, n (%)**			
	Artificial Neural Network	2 (5)	S8, S26
	Convolutional Neural Network	3 (8)	S10, S22, S24
	Deep Neural Networks	4 (11)	S14, S17, S21, S28
	Support Vector Machine	6 (16)	S4, S9, S25, S29, S30, S33
	Random Forest	7 (19)	S2, S5, S15, S18, S27, S36, S37
	Long Short-Term Memory	1 (3)	S13
	Decision Trees or Gradient Boosting Decision Trees	2 (5)	S31, S35
	K-Nearest Neighbors	1 (3)	S31
	Multilayer Perceptron	1 (3)	S6
	OneR	1 (3)	S7
	Ensemble	1 (3)	S1
	Support Vector Regression	1 (3)	S3
	Not mentioned	6 (16)	S11, S19, S20, S23, S32, S34

#### AI and ML Versus Wearables Versus Diabetes

**Figure 3:**
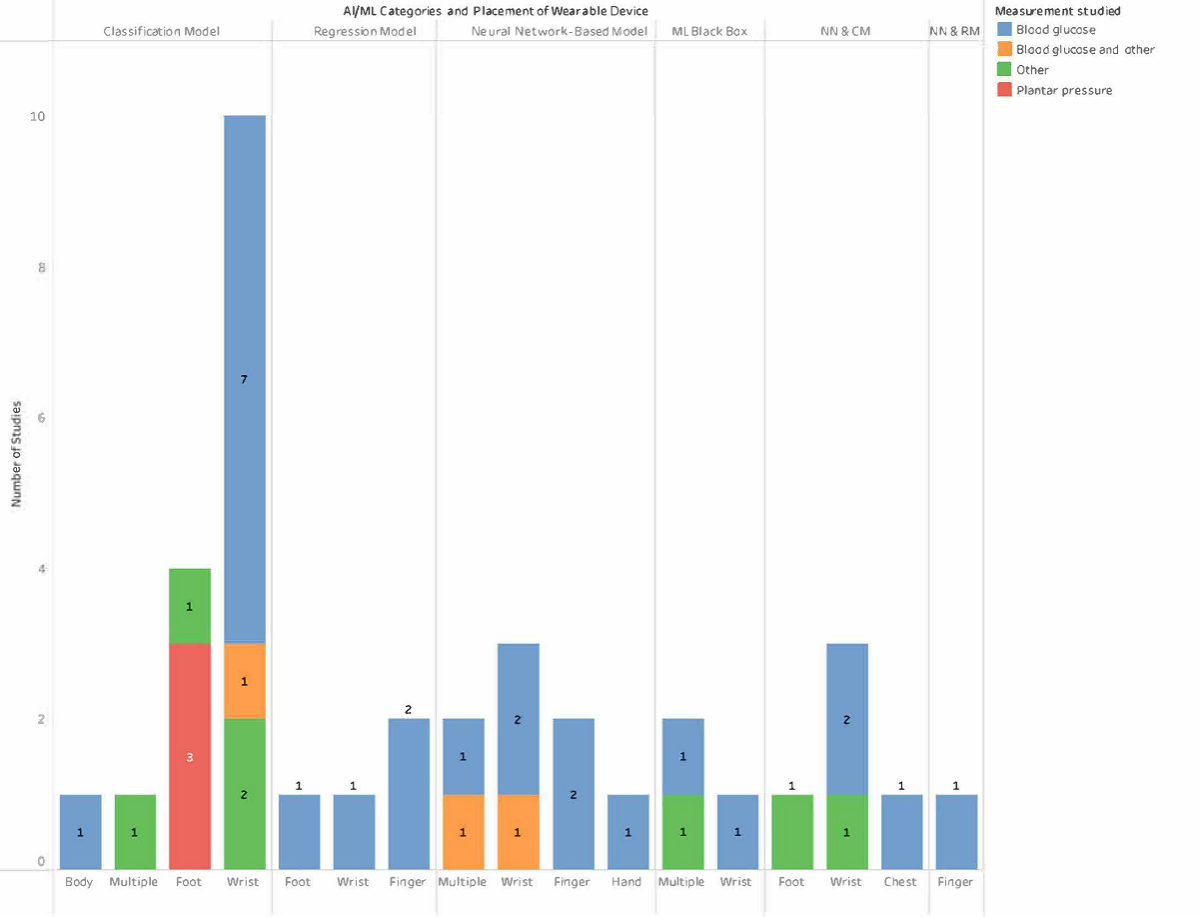
Artificial intelligence (AI) or machine learning (ML) models used with regard to wearable device placement and measurement studied. CM: classification model; NN: neural network; RM: regression model.

Figure 3 shows the category of the ML algorithm used according to each WD placement and measurement. Most devices that made use of classification models among the wrist-worn devices were the most prominent. Neural network and regression model were the least used.

## Discussion

### Principal Findings

This was the first study of its kind to the best of our knowledge, considering the amount of features we were able to extract from each publication. The features extracted should give researchers insight not only into the technologies that are readily available commercially but also into what is possible in the future with studies we identified that developed prototypes. Our findings shed light on this emerging field, which is still in its infancy. This is further highlighted by the fact that 59% (22/37) of the studies that met our inclusion criteria were prototypes; we were only able to identify 41% as commercially available (as demonstrated in Multimedia Appendix 6) devices, of which only (7/15, 46%) studies performed some sort of ML classification on the extracted data directly from WDs, whereas (6/15, 40%) studies made use of neural network–based models with classification to make out of already collected data. Most of these measured blood glucose on wrist-worn devices and used a classification algorithm (Figure 3). Classification models were widely used (Figure 3) in the reviewed studies, largely owing to studies attempting to classify types of diabetes (T1D, T2D, etc). SVM and random forest were the most prevalent classifiers and exhibited the highest performance. SVM [55] is extensively used because of its superiority in generalization and nonlinear function fitting, and it also has a number of advantages when dealing with small-sample studies [56]. Furthermore, SVM is a binary classifier, and we observed that it is mostly used on blood glucose level data to determine levels for diabetes categorization. Aside from the accuracy for demonstrating efficacy, the Clarke Error Grid was the most commonly used performance metric, possibly because of its popularity as a performance metric for assessing blood glucose estimation. The grid was split into 5 zones, each with varied prediction accuracy between the estimated and reference blood glucose readings. The data fell within zone A, which pertains to precise glucose calculations, where each consecutive zone is thought to have progressively substantial erroneous estimations [57,58]. Most of the sensory data being collected especially when looking at commercially available devices, did not require any further or minimal input from the user, meaning the person with diabetes can get on with day-to-day tasks without having to worry about taking regular invasive finger pricks for monitoring glucose levels; for example, while still feeling that they are wearing a stylish item such as a smartwatch. We specifically examined studies after 2015, as previous studies related to the use of WDs found that most wearables were used in this range [59]. One of the reasons may be that Fitbit released its first device in 2009 and the Apple Watch followed in 2015; both these devices set the tone for WDs, and it is not surprising that 59% (22/37) from our review were wrist-worn. A total of 78% (29/37) of the devices were connected to either a gateway or host device, usually a smartphone (16/37, 43%) via either Bluetooth (19/37, 51%) or Wi-Fi or internet (6/37, 16%); this is likely owing to the fact that web-based data are now more affordable and the availability of low energy connectivity technology such as Bluetooth. This ability to connect has resulted in more analytics and data storage being possible on host devices than on smartphones or directly on the cloud (18/37, 49%) of studies in this review, compared with limited computing power on the WD itself. One of the limitations of this is that devices need continuous connections, which can be an issue, as reported data can be lost if the connection is not maintained for long periods [60]. We also observed that many devices used gateways or host devices, which we believe to be largely because of the limited computing power of WDs.

### Strengths

This review was conducted according to the PRISMA-ScR; therefore, it can be considered a high standard. Two reviewers independently conducted the study selection and data extraction. We believe this to be the first of its kind study focusing on WDs targeting diabetes using AI approaches and were unable to identify previous scoping reviews in the literature that has as an exhaustive list of features extracted in this field. A combination of expert research computer scientists and research medical practitioners allowed us to explore the current technologies in depth and highlight gaps in the research community. The most popular databases in the health care and information technology fields were searched; furthermore, Google Scholar with forward and backward reference list checking allowed an exhaustive search of the literature, reducing the risk of publication bias.

### Limitations

Only studies published between 2015 and 2021 in the English language were included. Furthermore, we did not use Medical Subject Headings terms in our search; therefore, we may have overlooked some relevant studies. We excluded devices that could be classified as WDs, such as electroencephalogram and ECG machines, which limited their use in hospital settings. As our focus was AI, we excluded any study of WDs and diabetes that had a statistical measurement not considered an AI approach. Although we included a large number of features and some effectiveness measures, we fall short of critically assessing the quality of each of the included studies—this goes beyond the scope of our review—and we hope to cover this in a full systematic review in the near future on the same topic.

### Practical and Research Implications

WDs hold great potential for the self-monitoring of diabetes-related parameters, and their ability to be paired with a range of smart devices, including smartphones and general connectivity to clouds, allows the continuous collection of data from many biosensors that measure vitals and biosignals without user interference. The fact that they can be worn in a stylish and fashionable manner has potential for wider acceptance than other technologies, such as CGMs. Although many studies have used WDs for diabetes, we found that ML is still lacking in a sizable number of these studies. With the limited number of studies that reported the use of ML, we see great promise, largely owing to the accuracy levels of the ML algorithms reported in Table 6. Engineering and data science research experts need to come together and identify the most common sensors and technologies and study their effectiveness when combined with ML approaches. In addition, commercially available WDs are readily available and therefore sit in waiting for researchers to conduct studies and apply ML and report further in scientific journals to prove validity and instill consumer confidence. Most of the papers identified in this study used AI or ML algorithms for testing the validity of the system functioning rather than identifying the approaches that could be used for the development of such intelligent devices. More work needs to concentrate on applying known ML algorithms for the purpose of making more accurate diabetes-related measurement calculations. Currently, the number of commercial devices associated with studies are still very low, a quick search on retail sites such as Amazon reveals many commercial devices claiming diabetes-related measurements, which have still yet to be validated with related studies, and this is one area where researchers could get involved. Researchers need to make more use of purpose-shifted devices as they are lying in wait as opposed to creating prototypes and testing the effectiveness of the many commercially available devices. We encourage researchers to perform systematic reviews to assess the efficacy of AI-based and non–AI-based WDs compared with traditional medical devices. Some technologies that are classified as WDs such as CGMs are still classified as semi-invasive as they allow the embedding of a sensor partially into participants’ skin, we feel for wider acceptance especially for home use products the technology really needs to move away from such sensors and more studies now need to focus on how measurements can be obtained from noninvasive sensors such as those available on commercial smart watches. Further work is also required on ML algorithms used for diabetes data that can be used on the WDs as opposed to on host devices, as this would reduce some issues reported such as loss of data owing to WD out of range with the host device, which will become easier with time as the technology advances and WD memories are no longer a limitation. We suspect there would be less reliance on host devices for some of the ML computations.

Another area for exploration is the use of the internet of things (IoT); in our search, we found a handful of studies making use of IoT. Most IoT papers describe the IoT architecture for diabetes management without specifying the sensors or WDs actually used or implemented, and do not go into much (if any) detail about any ML deployed. There are many opportunities in this domain; none of the studies were found to make good use of developed commercial technologies such as Alexa, Google Home, and Apple watches, which are readily available. The possibilities here are endless, using a combination of data gathered from sensors at the WDs with other patients and personal data in real-time with IoT. This brings along with its own caveats and the need to incorporate questions of privacy and data sovereignty arising from the mass data storage in cloud-based systems and the many interconnected devices and hospital datacenters; there are issues that need to be considered with the use of data and individual consent. There are also problems regarding the scope of an individual’s consent to use their data, as well as potential accountability if the data are mishandled. There are dangers associated with AI algorithms and their misdiagnoses, dangerous advice, or recommendations that do not correspond to the required standard of care. Data security breaches or the reidentification of previously deidentified data may have unintended repercussions. Furthermore, other ethical issues need to be considered, such as accessibility, although commercial WDs that are easily and cheaply available may not be affordable for the masses in low-income countries. A multidisciplinary effort is required, including but not limited to engineers, medical practitioners, and legal experts.

### Conclusions

We investigated and reported the current state of WDs and their features for the purpose of diabetes that use ML approaches. Considering the availability of consumer-grade biosensors, we see great advancement potential in this domain, replacing hospital setting, invasive devices, especially when it comes to monitoring glucose levels. Further clinically significant studies are needed to instill confidence and validate WD use as well as the application of ML algorithms on WD data. Researchers and those wanting to develop AI-based WDs can use our review to understand where the gaps are in this emerging field. We encourage readers to use more data and delve deeper into the studies we have identified in order to establish, validate, and repeat studies that showed high accuracy. There is still much work needed, and we feel our review has provided the most extensive work so far summarizing WDs that use ML for people with diabetes to date. Finally, researchers will also benefit from our study as they can embark on longer and better populated systematic studies scrutinizing the benefits of WDs as data gathering, monitoring, prediction, classification, and recommendation devices in the context of diabetes. We envisage several follow-up studies, starting with a full systematic review from our own group.

## Appendix

Multimedia Appendix 1 PRISMA (Preferred Reporting Items for Systematic Reviews and Meta-Analyses) checklist.

Multimedia Appendix 2 Full search terms and strings table.

Multimedia Appendix 3 Data extraction form.

Multimedia Appendix 4 Study reference table.

Multimedia Appendix 5 Data extraction sheet.

Multimedia Appendix 6 Wearable technologies status with regard to wearable device technologies.

## Abbreviations

AI: artificial intelligence
CGM: continuous glucose monitoring
ECG: electrocardiogram
IoT: internet of things
ML: machine learning
OS: operating system
PRISMA-ScR: Preferred Reporting Items for Systematic Reviews and Meta-Analyses extension for Scoping Reviews
SVM: support vector machine
T1D: type 1 diabetes
T2D: type 2 diabetes
WD: wearable device
