# Supplementary material for: Overview of Artificial Intelligence–Driven Wearable Devices for Diabetes: Scoping Review
Source: J Med Internet Res. 2022 Aug 9;24(8):e36010. doi: 10.2196/36010 (PMC9399882; doi:10.2196/36010)
Supplement: Multimedia Appendix 2 [file jmir_v24i8e36010_app2.docx]

**Multimedia Appendix 2 Full search terms and strings table.**

| **Database** | Search terms |
| --- | --- |
| **MEDLINE** | (Artificial Intelligence OR Machine Learning OR Deep Learning OR Decision tree OR KNearest Neighbor* OR Support vector machine* OR Recurrent neural network* OR convolutional neural network* OR Artificial neural network* OR Naive Bayes OR Fuzzy Logic OR KMeans OR Random Forest OR LSTM OR autoencoder OR boltzmann machine OR deep belief network) AND (  wearable* OR smart watch* OR smart* OR smartwatch* OR fitness band* OR flexible band* OR wristband* OR smart insole* OR bracelet*) AND (Diabetic OR Diabetes)  **Search period (2015- 2021)** |
| **EMBASE** | (Artificial Intelligence OR Machine Learning OR Deep Learning OR Decision tree OR KNearest Neighbor* OR Support vector machine* OR Recurrent neural network* OR convolutional neural network* OR Artificial neural network* OR Naive Bayes OR Fuzzy Logic OR KMeans OR Random Forest OR LSTM OR autoencoder OR boltzmann machine OR deep belief network) AND (  wearable* OR smart watch* OR smart* OR smartwatch* OR fitness band* OR flexible band* OR wristband* OR smart insole* OR bracelet*) AND (Diabetic OR Diabetes)  **Search period (2015- 2021)** |
| **PsycINFO** | (Artificial Intelligence OR Machine Learning OR Deep Learning OR Decision tree OR KNearest Neighbor* OR Support vector machine* OR Recurrent neural network* OR convolutional neural network* OR Artificial neural network* OR Naive Bayes OR Fuzzy Logic OR KMeans OR Random Forest OR LSTM OR autoencoder OR boltzmann machine OR deep belief network) AND (  wearable* OR smart watch* OR smart* OR smartwatch* OR fitness band* OR flexible band* OR wristband* OR smart insole* OR bracelet*) AND (Diabetic OR Diabetes)  **Search period (2015- 2021)** |
| **IEEE Xplore** | ("Artificial Intelligence" OR "Machine Learning" OR "Deep Learning" OR "Decision tree" OR "Recurrent neural network*" OR "convolutional neural network*" OR "Artificial neural network*") AND("wearable*" OR “smart watch*” OR “fitness band*” OR “flexible band*” OR "wristband*" OR “smart*” OR “bracelet*”) AND ("Diabetic" OR "Diabetes")  **Search period (2015- 2021)** |
| **ACM Digital Library** | ("Artificial Intelligence" OR "Machine Learning" OR "Deep Learning" OR "Decision tree" OR "K-Nearest Neighbor*" OR "Support vector machine*" OR "Recurrent neural network*" OR "convolutional neural network*" OR "Artificial neural network*" OR "Naïve Bayes" OR "Naive Bayes" OR "Fuzzy Logic" OR "K-Means" OR "Random Forest" OR “LSTM” OR “autoencoder” OR “boltzmann machine” OR “deep belief network”) AND (“wearable*” OR “smart watch*” OR “smart*” OR smartwatch* OR “fitness band*” OR “flexible band*” OR “wristband*” OR “smart insole*” OR “bracelet*”) AND (“Diabetic” OR “Diabetes”)  **Search period (2015- 2021)** |
| **Google Scholar** | ("Artificial Intelligence" OR "Machine Learning" OR "Deep Learning" OR "convolutional neural network*" OR "Artificial neural network*") AND (wearable*” OR “smart watch*” OR “smart*”) AND (“Diabetic” OR “Diabetes”)  **Search period (2015- 2021)** |
| **Web Of Science** | ("Artificial Intelligence" OR "Machine Learning" OR "Deep Learning" OR "Decision tree" OR "K-Nearest Neighbor*" OR "Support vector machine*" OR "Recurrent neural network*" OR "convolutional neural network*" OR "Artificial neural network*" OR "Naïve Bayes" OR "Naive Bayes" OR "Fuzzy Logic" OR "K-Means" OR "Random Forest" OR “LSTM” OR “autoencoder” OR “boltzmann machine” OR “deep belief network”) AND (“wearable*” OR “smart watch*” OR “smart*” OR smartwatch* OR “fitness band*” OR “flexible band*” OR “wristband*” OR “smart insole*” OR “bracelet*”) AND (“Diabetic” OR “Diabetes”)  **Search period (2015- 2021)** |
