# Supplementary material for: Overview of Artificial Intelligence–Driven Wearable Devices for Diabetes: Scoping Review
Source: J Med Internet Res. 2022 Aug 9;24(8):e36010. doi: 10.2196/36010 (PMC9399882; doi:10.2196/36010)
Supplement: Multimedia Appendix 3 [file jmir_v24i8e36010_app3.docx]

**Multimedia Appendix 3. Data extraction form.**

| **Features Name** | **Description** | **Example** |
| --- | --- | --- |
| **Age range of participants** | What age of participants are used for investigation in study |  |
| **Accuracy** | Evaluation of Models performance |  |
| **AI or ML technologies used** | what AI or ML algorithms are used | Machine Learning and Ensemble Learning, Convolutional Neural Network, Long Short-term Memory etc. |
| **Application of AI technology used** | what specific purpose the technology is used for in study | CNN (Diabetes detection), CNN and LSTM- RNN (Blood pressure monitoring) |
| **Collected data for analysis Source** | How data is collected for the device making |  |
| **Country** | Which country the article is published from |  |
| **Device Technology** | what's the commercial technology used | Fitbit, mi band, apple watch, Digi-Walker Pedometer etc |
| **Diabetes type studied** | what type of diabetes is under consideration in the paper | Type 1, Type 2 etc. |
| **Host device** | what is the end gate device the wearable is synchronised with | mobile, tablet, ipad, laptop,computer. |
| **Mode of Data transfer** | how data is transferred from wearable device to host device | WIfi, Bluetooth, wired, GPS, Airdrop, mobile data |
| **Number of participants** | how many participants used in the experiment – clearly specify the healthy, unhealthy, mail, female separately |  |
| **Operating systems** | what operating systems it supports or compatible with | Andrioid, IOS, None or both |
| **Placement of wearable device** | where the wearable device is worn during the case study/experiment in paper or normally | hands, ankles, forehead, forearms, eyes, fingers etc. |
| **Primary measure of device** | What are the primary data measured by the device | heart rate, skin conductance etc |
| **Privacy and security** | what privacy and security measures are adopted by the wearables for data protection |  |
| **Publication Type** | What type of article it is | Journal article, Conference proceedings |
| **Publisher** | Which database the article is published in |  |
| **Sensing Approach** | how data is being collected by the wearables with or without knowledge of user | participatory approach (manually and non-intrusive), opportunistic approach (automatic mechanisms for collecting data). |
| **Sensing Technology** | sensor technology used to capture motor and metabolic health information via users’ physical performance and contact with the skin | photosensors, ACC (acceleration), Gyroscope (angular velocity), NIR (Near infrared) etc |
| **Study aim** | what is study aim in regards to diabetes monitoring through wearables | prevention, risk assessment, self-management (calculation of how much insulin (if using) dosage needed), monitoring (the glucose levels only) - self-administer |
| **Study conducted Duration** | How long it took to conduct the experiment |  |
| **Wearable Device Type** | what type of device it is | smart band, smart watch, smart glasses, smart clothes etc |
| **Wearable technology status** | is the wearable device a prototype or is already available commercial device (Fitbit, apple watch) | Prototype, Commercial |
| **Year** | What year the study was published |  |
| **Other key observation** | related to data manipulation, processing, or other technological terms, data related |  |
