# Supplementary material for: Overview of Artificial Intelligence–Driven Wearable Devices for Diabetes: Scoping Review
Source: J Med Internet Res. 2022 Aug 9;24(8):e36010. doi: 10.2196/36010 (PMC9399882; doi:10.2196/36010)
Supplement: Multimedia Appendix 4 [file jmir_v24i8e36010_app4.docx]

**Multimedia Appendix 4. Study reference table.**

| **Study ID.** | **Study title** | **Reference** |
| --- | --- | --- |
| S1 | 5G-Smart Diabetes: Toward Personalized Diabetes Diagnosis with Healthcare Big Data Clouds | [22] |
| S2 | A mobile system for sedentary behaviors classification based on accelerometer and location data | [23] |
| S3 | A Noninvasive Glucose Monitoring SoC Based on Single Wavelength Photoplethysmography | [24] |
| S4 | A Noninvasive, Economical, and Instant-Result Method to Diagnose and Monitor Type 2 Diabetes Using Pulse Wave: Case-Control Study. | [25] |
| S5 | A Novel Low-Cost Wireless Footwear System for Monitoring Diabetic Foot Patients | [26] |
| S6 | A Personalized Healthcare Monitoring System for Diabetic Patients by Utilizing BLE-Based Sensors and Real-Time Data Processing | [27] |
| S7 | A Smart Glucose Monitoring System for Diabetic Patient | [28] |
| S8 | An IoT-Based Non-Invasive Glucose Level Monitoring System Using Raspberry Pi | [29] |
| S9 | Classification-Based Screening of Phlebopathic Patients using Smart Socks | [30] |
| S10 | Design and Implementation of a Wearable System for Non-Invasive Glucose Level Monitoring | [13] |
| S11 | Design of a Non-invasive ECG-based Glucose Measurement System | [31] |
| S12 | Design of intelligent diabetes mellitus detection system using hybrid feature selection based XGBoost classifier | [32] |
| S13 | Determining Physical Activity Characteristics From Wristband Data for Use in Automated Insulin Delivery Systems | [33] |
| S14 | DiabDeep: Pervasive Diabetes Diagnosis Based on Wearable Medical Sensors and Efficient Neural Networks | [34] |
| S15 | Diabetes Care in Motion: Blood Glucose Estimation Using Wearable Devices | [35] |
| S16 | Dia-Shoe: A Smart Diabetic Shoe to Monitor and Prevent Diabetic Foot Ulcers | [36] |
| S17 | iGLU 2.0: A New Wearable for Accurate Non-Invasive Continuous Serum Glucose Measurement in IoMT Framework | [37] |
| S18 | Joint Empirical Mode Decomposition and Singular Spectrum Analysis Based Pre-processing Method for Wearable Non-invasive Blood Glucose Estimation | [38] |
| S19 | Joint empirical mode decomposition, exponential function estimation and L-1 norm approach for estimating mean value of photoplethysmogram and blood glucose level | [39] |
| S20 | Live Demonstration: A Single LED PPG-Based Noninvasive Glucose Monitoring Prototype System | [40] |
| S21 | Multi-Modal Predictive Models of Diabetes Progression | [7] |
| S22 | Nocturnal low glucose detection in healthy elderly from one-lead ECG using convolutional denoising autoencoders | [41] |
| S23 | Non-invasive Analytics Based Smart System for Diabetes Monitoring | [42] |
| S24 | Non-invasive Blood Glucose Estimation Using Multi-sensor Based Portable and Wearable System | [43] |
| S25 | Non-invasive Diabetes Mellitus Detection System using Machine Learning Techniques | [8] |
| S26 | Non-invasive monitoring of blood glucose by means of wearable tracking technology | [44] |
| S27 | Non-invasive wearables for remote monitoring of HbA1c and glucose variability: Proof of concept | [45] |
| S28 | PPG-Based Smart Wearable Device With Energy-Efficient Computing for Mobile Health-Care Applications | [14] |
| S29 | Prediction of Diabetic Peripheral Neuropathy (DPN) using Plantar Pressure Analysis and Learning Models | [46] |
| S30 | Smart Wristband with Integrated Chemical Sensors for Detecting Glucose Levels using Breath Volatile Organic Compounds | [47] |
| S31 | SmartSock: A Wearable Platform for Context-Aware Assessment of Ankle Edema | [48] |
| S32 | SMEAD: A secured mobile enabled assisting device for diabetics monitoring | [49] |
| S33 | The Internet of Things and Big Data Analytics for Chronic Disease Monitoring in Saudi Arabia. | [50] |
| S34 | The Promise and Perils of Wearable Physiological Sensors for Diabetes Management | [54] |
| S35 | Towards Wearable-Based Hypoglycemia Detection and Warning in Diabetes | [51] |
| S36 | Transformation of Temperature Timeseries into Features that Characterize Patients with Diabetic Autonomic Nerve Disorder | [52] |
| S37 | Wearable Non-invasive Blood Glucose Estimation via Empirical Mode Decomposition Based Hierarchical Multiresolution Analysis and Random Forest | [53] |
