# Supplementary figures and images for: Overview of Artificial Intelligence–Driven Wearable Devices for Diabetes: Scoping Review
Source: J Med Internet Res. 2022 Aug 9;24(8):e36010. doi: 10.2196/36010 (PMC9399882; doi:10.2196/36010)

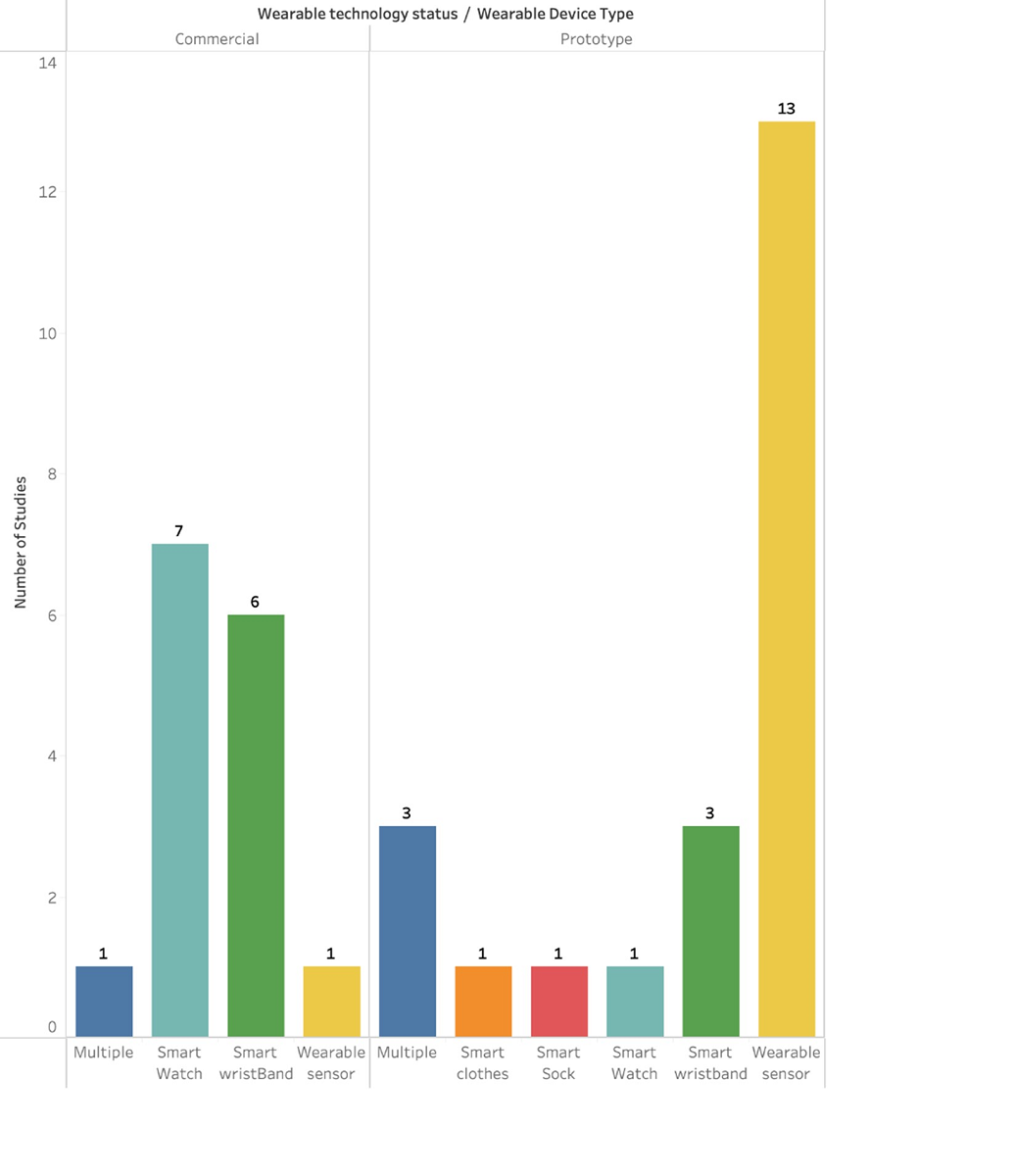

Supplement: Multimedia Appendix 6 [file jmir_v24i8e36010_app6.png]
